# Current Management of Resistant Hypertension in Patients with Intracerebral Hemorrhage

**DOI:** 10.3390/ijms27062716

**Published:** 2026-03-16

**Authors:** Michelle Nguyen, Sookyung Oh, Matthew King, Wengui Yu, Ahmad Riad Ramadan

**Affiliations:** 1 Department of Neurology, University of California Irvine, Orange, CA 92868, USA; 2 Department of Cardiology, University of California Irvine, Orange, CA 92868, USA

**Keywords:** intracerebral hemorrhage, resistant hypertension, stroke

## Abstract

Approximately 795,000 people experience new or recurrent strokes in the United States each year; between 10 to 20% of these are spontaneous intracerebral hemorrhages (ICH). Uncontrolled hypertension is not only the most common cause of ICH but also a major risk factor for hematoma expansion. Resistant hypertension, defined as persistently elevated blood pressure despite the use of three or more antihypertensives of different classes, is common in patients with ICH. A long-acting calcium channel blocker, angiotensin-converting enzyme inhibitor (ACEi) or angiotensin receptor blocker (ARB), and a thiazide diuretic are generally considered the mainstay for the treatment of resistant hypertension. However, due to the risk of hyponatremia and worsening cerebral edema, thiazide diuretics should be avoided during the first few weeks of ICH. Recent evidence supports the use of a mineralocorticoid receptor antagonist. While resistant hypertension may be idiopathic, a workup of secondary causes should be pursued. Adequate and timely control of elevated blood pressure remains one of the main cornerstones of treatment in patients with ICH. Previous studies have revealed that resistant hypertension in patients with ICH is associated with longer ICU stays, a higher risk of recurrent stroke, and can contribute to renal, cardiac, and neurologic complications. This emphasizes the need for early initiation of oral antihypertensives and adequate blood pressure control at hospital discharge. Landmark studies have shown that early lowering of SBP to 130–150 mm Hg with smooth, sustained BP control is safe and may improve functional outcomes in patients with mild to moderate ICH. After initiating oral antihypertensives with a calcium channel blocker, an ACEi or ARB beta-blocker, and a mineralocorticoid receptor antagonist to maximally tolerated doses, the next line of antihypertensive treatment should be tailored to the patient’s co-morbidities, and may include a beta-blocker, central alpha agonist, hydralazine, and minoxidil. In this review, we discuss the epidemiology of resistant hypertension in ICH and its molecular basis, diagnostic workup, and acute and long-term treatment. We present novel mechanisms implicated in hypertensive ICH, including ferroptosis, neuroinflammation, the CNS–gut microbiome axis, and novel therapeutics. We also propose a simple algorithm for the optimal pharmacological management of resistant hypertension in ICH.

## 1. Introduction

**Figure 1:**
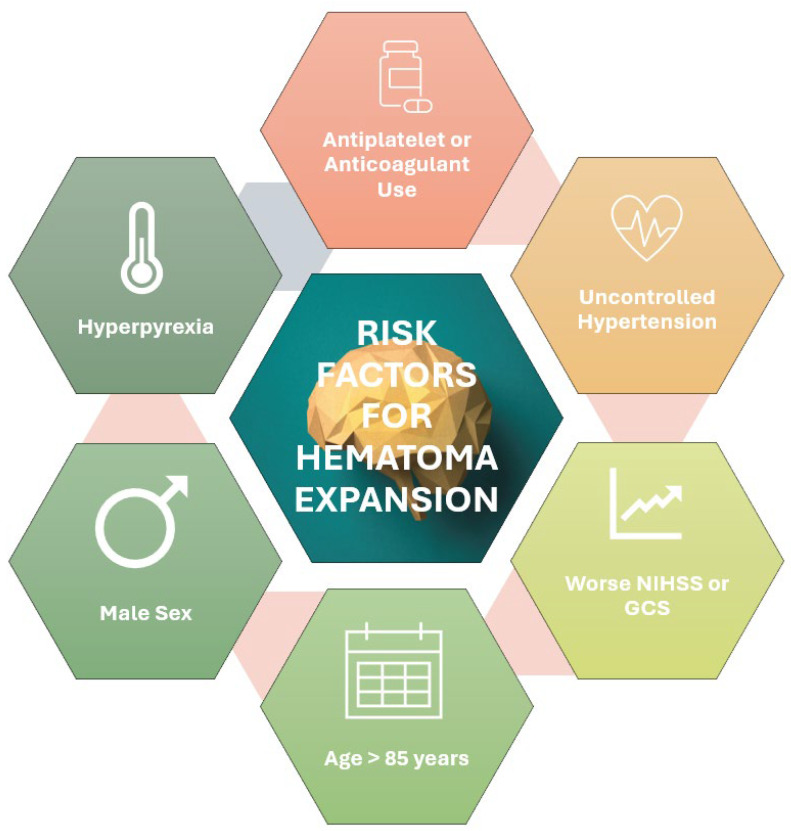
Risk factors for hematoma expansion include uncontrolled hypertension, age > 85 years, male sex, increased severity of presenting symptoms (NIHSS/GCS), antiplatelet or anticoagulant use, and hyperpyrexia. GCS: Glasgow Coma Scale; NIHSS: National Institutes of Health Stroke Scale.

Approximately 795,000 people experience new or recurrent strokes each year in the United States; approximately 10 to 20% of these are intracereberal hemorrhages [1,2,3]. Intracerebral hemorrhage (ICH) may be primary (due to small-vessel disease in the setting of hypertension and cerebral amyloid angiopathy) or secondary to a lesion or bleeding diathesis [4]. Hematoma expansion in intracerebral hemorrhage is associated with worse outcomes [5]. Clinical predictors of hematoma expansion include antiplatelet or anticoagulant use, higher baseline NIHSS or lower GCS, hyperpyrexia, and alcohol use [6]. Individuals older than 85 years of age and of male sex are also at higher risk of hematoma expansion [6]. However, uncontrolled hypertension is not only one of the strongest risk factors for ICH but also a major contributor to hematoma expansion [7]. Therefore, the hypertensive patient with ICH requires prompt attention (Figure 1).

Clinically, hypertension is defined as a systolic blood pressure greater than 130 mmHg, or a diastolic blood pressure greater than 80 mmHg [8]. Uncontrolled hypertension is commonly attributed to resistant hypertension, which is defined as persistently elevated blood pressure despite the use of three or more antihypertensives of different classes, such as a long-acting calcium channel blocker, ACE/ARB, and a thiazide-like diuretic [9,10,11].

Globally, an estimated 100–500 million people are affected by resistant hypertension; a previous meta-analysis revealed that resistant hypertension has a prevalence of 5% amongst patients with hypertension [12]. It is more often seen in male patients, those with diabetes mellitus, and those of black African descent [12]. A study published by our group found that 31.6% of patients with hypertensive ICH had resistant hypertension.

Previous studies have revealed that resistant hypertension in patients with ICH is associated with longer lengths of stay in the ICU, as well as greater requirements for ventilator support, hematoma evacuation, hypertonic saline therapy, and nicardipine infusion [13]. The risk of recurrent stroke is also higher in survivors of ICH with resistant hypertension at 3 months post ICH [8]. Furthermore, refractory hypertension can contribute to systemic disease (as with renal and cardiac injury) and may result in posterior reversible encephalopathy syndrome (PRES), hypertensive encephalopathy, and intraocular hemorrhage. These complications emphasize the need for early initiation of oral antihypertensives and adequate blood pressure control at discharge in patients with ICH.

In this paper, we first review the molecular basis of benign and resistant hypertension, highlighting the most recent advances in pathogenesis but also identifying gaps in knowledge and future directions that the field is taking. We then discuss the acute management of hypertension in patients with ICH, detailing the various classes of antihypertensive medications, followed by the recommended diagnostic workup of secondary causes of hypertension. We explore novel therapeutics and research, highlighting translational research on animal models of resistant hypertension. Finally, we address considerations for the long-term management of hypertension in patients with ICH and discuss bundle therapy with triple or quadruple antihypertensives, targeting multiple molecular pathways in future clinical research.

## 2. Molecular Mechanisms in Hypertension

The mechanisms underlying hypertension are the result of a complex interplay between environmental, genetic/epigenetic, neural, endocrine, and inflammatory factors [14]. Chronic overactivation of the renin–angiotensin–aldosterone system (RAAS), tonic mineralocorticoid receptor signaling, and heightened sympathetic nervous system activity form the basis of resistant hypertension in ICH. Together, these factors culminate in vascular remodeling, fluid overload, oxidative stress, and endothelial dysfunction [15].

KEGG (Kyoto Encyclopedia of Genes and Genomes) pathway enrichment analysis has allowed for the identification of differentially expressed genes (DEGs) and molecular pathways that are central to the development of resistant hypertension and its role in ICH. Using multi-omic data, KEGG can integrate large amounts of genomic, chemical, and functional information to generate pathway maps and diagrams that help to describe the pathogenesis of specific conditions. In hypertensive ICH, for instance, KEGG has identified multiple pathways linked to vascular remodeling. Some of these pathways include: HIF-1, MAPK, AMPK, PI3K-AKT, RAGE, TGF-β, NF-kappa B, and JAK-STAT. Together, these pathways lead to intense immune/inflammatory upregulation and vascular remodeling through the interplay of excessive collagen and calcium deposition in the extracellular matrix (ECM), as well as vascular smooth muscle cells (VSMCs) proliferation, and phenotypic switch to osteoblast/chondroblast-like cells, culminating in vessel wall mineralization and arterial stiffness [16].

### 2.1. Endothelial Dysfunction and Oxidative Stress

The cell signaling underlying vascular function primarily involves an interplay between the RAAS, calcium signaling, the nitric oxide–nitric oxide sensitive guanylate cyclase–cGMP pathway (NO–NOsGC–cGMP), and vascular remodeling. Calcium and NO–NOsGC–cGMP signaling are reversible, while vascular remodeling is less so.

The RAAS is the master hormonal system responsible for maintaining hemodynamic physiology by modulating vascular tone, sodium/potassium homeostasis, and circulating fluid volume. Renin is stored in the juxtaglomerular cells of the afferent arterioles of the kidney, and is released following reductions in renal perfusion, the activation of beta-1 adrenergic receptors, increased sodium/chloride concentration at the distal convoluted tubule, and hypokalemia [17]. It catalyzes the cleavage of hepatic angiotensinogen to angiotensin I (Ang I). Angiotensin I does not have a known biological activity and is converted by the angiotensin-converting enzyme (ACE) to angiotensin II (Ang II). Ang II is the main effector for blood pressure elevation by stimulating the contraction of vascular smooth muscle, aldosterone secretion from the zona glomerulosa of the adrenal cortex, and the release of vasopressin from the hypothalamus. It also causes increased sodium resorption and increased sympathetic outflow. Another important effector of RAAS, the mineralocorticoid aldosterone acts on the late distal tubule and collecting duct of the nephron, resulting in sodium and water reabsorption, and potassium excretion, ultimately leading to blood pressure elevation [18].

When stimulated, vascular smooth muscle cells release intracellular stores of calcium and extracellular calcium enters through high-voltage activated L type calcium channels. This results in an increased concentration of intracellular calcium. Calcium binds to calmodulin; this complex activates myosin light-chain kinase, which phosphorylates the myosin light chains promoting contraction [15].

Activation of the NO–NOsGC–cGMP pathway promotes vasodilation. Nitric oxide is produced in endothelial cells and RBCs and diffuses to vascular smooth muscle cells. It then binds to soluble guanylyl cyclase, which then produces cyclic GMP. Higher concentrations of cGMP result in the activation of protein kinase G and phosphorylation of vasodilator-stimulated phosphoprotein. Subsequently, myosin light-chain phosphatase de-phosphorylates contracted, phosphorylated actin-myosin, resulting in relaxation and vasodilation. NO–NOsGC–cGMP signaling also decreases renin release and promotes natriuresis by modulating sodium transporters [19].

Hypertension is known to contribute to the development of atherosclerotic lesions and promote vascular remodeling, ultimately resulting in the reduced elasticity of blood vessels. Based on Glagov’s phenomenon, the arteries remodel to maintain constant flow despite disruption from atherosclerotic mass lesions [20]. The formation of atherosclerotic plaque, in turn, results in disturbed blood flow and shear stress, thereby promoting pro-atherogenesis through NFkB and MAPK signaling.

**Figure 2:**
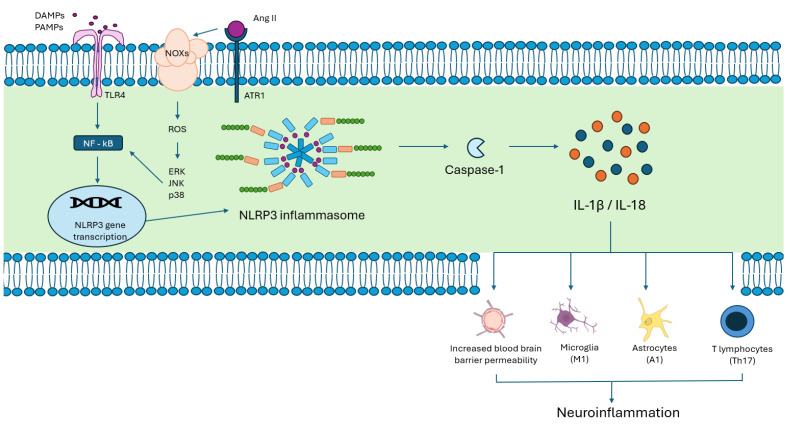
Activation of the NLRP3 inflammasome either via binding of DAMPs or PAMPs, such as lipopolysaccharide (LPS), to TLR4 receptors results in increased blood–brain barrier permeability and neuroinflammation, mediated by the activation of microglia, astrocytes, T lymphocytes, and endothelial cells by IL-1b and IL-18 via Caspase-1 activation. Upregulation of this neuroinflammatory cascade can also happen through RAAS activation and the binding of Ang II to ATR1. This illustrates the effect that RAAS activation has on neuroinflammation. DAMPs: damage-associated molecular patterns; NLRP3: NOD-like receptor family pyrin domain containing 3; NOX: NADPH oxidase; PAMPs: pathogen-associated molecular patterns, ROS: reactive oxygen species; TLR4: toll-like receptor 4.

NFkB is a transcription factor found in the cytoplasm as a homo- or hetero-dimer that is bound to IkB and controls the expression of pro-inflammatory gene products in association with cancer (breast, ovarian) as well as obesity, insulin resistance, and atherosclerosis [21]. Once stimulated (for instance, by disturbed blood flow/shear stress) the IkB inactivators are ubiquitinated by E3 ubiquitin ligase and degraded by proteasomes. NFkB translocates to the nucleus and results in the transcription of a multitude of gene products, including matrix metalloproteinases (MMPs), pro-inflammatory cytokines (IL-1, TNF alpha), and VEGF (Figure 2).

Consequent vascular remodeling occurs primarily because of excessive collagen production, advanced glycation end-product (AGEs) deposition, degradation of elastic fibers, and calcification within the extracellular matrix. Within the extracellular matrix, MMPs are responsible for degrading collagen and gelatin; however, some forms may also promote calcification, collagen accumulation, and elastin fiber degradation, ultimately resulting in increased arterial stiffness. AGEs may accumulate due to diabetes, hyperlipidemia, and smoking. AGEs may deposit in the extracellular matrix and form cross-links, which results in the deposition of other macromolecules. Collagen fibers cross-linked to AGEs are stiffer and contribute to vascular dysfunction. Medial arterial calcification and the resultant reduced vascular compliance are hypothesized to occur as the result of a loss of mineralization inhibition and induction of osteogenesis [15].

### 2.2. Novel Molecular Mechanisms Implicated in Hypertensive ICH

Cell death pathways, neuroinflammatory cascades, and the role of the gut–brain axis in hypertensive patients at risk of ICH are on the forefront of research efforts aimed at discovering therapies to dampen the secondary brain injuries majorly responsible for unfavorable clinical outcomes.

Ferroptosis, an iron-dependent, non-apoptotic cell death, has been linked to hypertensive cerebral injury. Hypertensive rats show a significant decrease in glutathione (GSH) and glutathione peroxidase 4 (GPX4) in the cerebral cortex [22]. GSH and GPX4 are the primary antioxidant enzymes that protect cells against lipid peroxidation. This process results in the accumulation of lethal lipid peroxides, which cause cell death by destroying cellular membranes. Ferroptosis is not unique to the brain, as it can also be seen in cardiomyocytes, proximal tubular epithelial cells, renal fibroblasts and podocytes, as well as pulmonary arterial endothelial cells, promoting hypertrophy and fibrosis in those target organs and contributing to various conditions such as heart failure, chronic kidney disease, and pulmonary hypertension. In mice models of ICH, lipid peroxidation inhibitors, ferrostatin-1 and liproxstatin-1, reduced hematoma size, cognitive and motor impairment [21]. Trials of ferroptosis inhibitors in ICH remain, however, in the preclinical phase.

Neuroinflammation is another pivotal process that plays a role in pathogenesis, drug discovery, and prognosis in ICH. The NLRP3 inflammasome, a cytosolic protein complex abundantly found in microglia, astrocytes and neurons, is activated by several stimuli such as released heme products and reactive oxygen species (ROS). The activation of the NRLP3 inflammasome leads to pyroptosis, an inflammatory form of programmed cell death, where a cell forms pores in its own cell membrane, lysing and releasing pro-inflammatory cytokines IL-1β and IL-18. The NRLP3 inflammasome also polarizes astrocytes, microglia, and lymphocytes to pro-inflammatory A1, M1 and Th17 phenotypes, respectively, perpetuating the inflammatory cycle, rendering the BBB more porous and exacerbating white matter injury [23]. This neuroinflammatory cascade culminates in neuronal and glial cell death, contributing to the poor outcomes seen after ICH. It is important to note, however, that NLRP3 inflammasome activation in the brain, particularly within the hypothalamic paraventricular nucleus (PVN) and microglia, occurs in the hypertensive brain, even in the absence of any ICH, triggering neuroinflammation, increased sympathetic outflow, and oxidative stress. NLRP3 inflammasome inhibitors (e.g., MCC950, MitoQ or baicalein) have shown reductions in white matter injury and cerebral edema in preclinical models of ICH [24].

While the CNS–gut microbiome axis has been investigated in a plethora of conditions, including cardiovascular diseases and ischemic strokes, scant data exist on the link between intestinal dysbiosis and ICH. Animal studies on mice models of ICH showed that inducing ICH in the striatum of mice caused gut dysmotility, gut wall dystrophy, and intestine–blood-barrier leakiness [25]. Furthermore, fecal matter transplants from healthy mouse donors decreased neuroinflammatory cytokines and improved neurobehavioral function after ICH. Importantly, several activators of the NRLP3 inflammasome originate from intestinal dysbiosis such as microbial lipopolysaccharide (LPS) binding to toll-like receptor 4 (TLR4). Conversely, activation of the NLRP3 inflammasome can influence the composition of the gut microbiome. Circulating cytokines and pro-inflammatory metabolites manage to cross permeable intestine–blood and blood–brain barriers, ultimately exerting their deleterious effects on the CNS. Therefore, manipulating the gut microbiome via administering fecal matter transplant, pre/probiotics or antibiotics could skew the profile of the gut microbiome to a pro- or anti-inflammatory response in the injured brain, depending on which microbial populations are targeted and what specific cytokines and metabolites are released [26].

There is mounting evidence that, beyond its effect on vascular physiology and renal homeostasis, the RAAS interplays significantly with the immune system through Ang II-mediated effects [27,28]. In fact, upon binding to Ang II, AT1R activation promotes inflammation by triggering mitochondrial ROS production, which acts as a key signal for NLRP3 inflammasome activation. Downstream effects of the crosstalk between Ang II-ATR1 and NLPR3 include pro-inflammatory cytokine production (TNF-α, IL-1β, IL-6, and IFN-γ), macrophage activation and endothelial adhesion molecules upregulation (ICAM-1, VCAM-1, and E-selectins). As such, RAAS inhibitors, such as ACE inhibitors (ACEIs) and angiotensin receptor blockers (ARBs), have been shown to play a significant immunomodulatory role, mostly through a reduction in pro-inflammatory cytokine production and the promotion of anti-inflammatory mediators [29]. A recent paper demonstrates this class benefit in patients with non-lobar ICH attributable to hypertensive arteriopathy, where the initiation of ACEIs or ARBs during hospitalization was associated with greater odds of favorable outcomes at 90 days (modified Rankin score of 0–2) compared to other antihypertensive classes such as thiazide diuretics, beta blockers and calcium channel blockers (adjusted OR, 1.49; 95% CI, 1.08–2.05; *p*  = 0.01) [30].

### 2.3. Acute Management of Hypertension in ICH Patients

Around 80% of patients with acute ICH present with elevated blood pressure. Persistently uncontrolled blood pressure is a risk factor for hematoma expansion, which has been associated with worse functional outcomes [31]. Two landmark RCTs, INTERACT2 and ATACH2 [32], were aimed at establishing whether aggressive blood pressure control could prevent hematoma expansion and improve mortality and morbidity after spontaneous ICH. INTERACT2 [33] was an international, multi-center, prospective, randomized, open-treatment, blinded end-point trial that compared outcomes with the early lowering of SBP to <140 mmHg or to <180 mmHg. The IV therapies and oral agents administered to achieve these goals were based on prespecified treatment protocols based on the local availability of agents; all patients received oral antihypertensives or topical nitrates within 7 days of the index event. Based on supplementary data provided by the INTERACT2 investigators, patients in the intensive BP-lowering group could receive hydralazine 5 to 20 mg IV every 5 min, metoprolol 5 mg IV every 3 to 5 min, topical glyceryl trinitrate 5 to 10 mg every 24 h, hydralazine infusion of 50 to 150 ug/min, labetalol 10 mg every 5 min, labetalol infusion 2–8 mg/min to max 300 mg/24 h, urapidil 5 mg every 5 min, urapidil infusion 5–30 mg/h, diltiazem 10 mg every 15 min, nimodipine 50 mL/50 mg at a rate of 4 mL/h, and furosemide 20–80 mg every 2 h for max of 1 g/day. No data were provided regarding the optimal dosing regimen at the conclusion of the trial. There was no significant difference in adverse events in the aggressive early lowering of SBP; upon ordinal analysis of mRS scores, intensive early lowering of SBP was associated with improved functional outcomes at 90 days. Interestingly, early intensive lowering of SBP did not clearly reduce hematoma growth; on subgroup analysis, there was no significant difference between patients who were randomized to treatment within 4 h of ICH and those who were randomized later. It was unclear if this was due to limited statistical power, or if the results truly represented an independent effect of early/later lowering of SBP from the time of event. It was hypothesized that intensive SBP lowering could be neuroprotective or reduce perihematomal edema with resultant downstream positive clinical outcomes.

ATACH II was a randomized, multi-center, two-group, open-label trial that studied the role of very early and aggressive reduction in patients with acute ICH presenting with SBP 170–200 mmHg. Patients were randomized to standard treatment or intensive treatment within 4.5 h of symptom onset. Using nicardipine infusion, SBP was maintained around 140–179 mmHg in the standard treatment group, and 110–139 in the intensive treatment group for 24 h after randomization. While there was no significant difference in neurologic deterioration at 24 h post randomization, nor in death or disability at 3 months, the intensive treatment group had a higher incidence of adverse renal events (9.0%, 4.0%, *p* = 0.002).

The differing conclusions reached by the two trials stem in large part from differences in BP management protocols with ATACH2 enrolling patients with higher average SBP and achieving the BP targets faster and more frequently than INTERACT2. Importantly, the achieved BP goal in ATACH2 was lower than that in INTERACT2, contributing to a larger reduction in BP. Based on the results of these trials, AHA guidelines state that early lowering of SBP to a target of 130–150 mm Hg, with smooth, sustained BP control using regimens that limit BP variability, is safe and may improve functional outcome in patients with mild to moderate acute ICH who present with SBP between 150 to 220 [34].

Post hoc analysis of INTERACT2 data revealed that the elevation of baseline SBP, glucose, body temperature, and anticoagulant use were independent predictors of a poor functional outcome post ICH. Therefore, INTERACT3 was designed to study the impact of bundled care on outcomes in ICH. The results of INTERACT3 showed that bundled care, with early intensive lowering of SBP (<140 mmHg), strict glucose control, avoidance of fever (≤37.5 °C), and reversal of anticoagulation within 1 h, improves functional outcomes in patients with acute ICH. While there was no significant difference in death or disability at 90 days, patients who received bundled care had better mRS scores and lower odds of death at 6 months. Therefore, while early and aggressive blood pressure management is paramount, anticoagulation reversal and the avoidance of hyperglycemia/fever is also crucial.

It was previously thought that aggressive blood pressure control in the acute period could result in perihematomal ischemia. The RCT, ICH ADAPT, showed that greater blood pressure reduction (SBP <150 mmHg vs. <180 mmHg) did not result in greater perihematomal ischemia or infarction. Along the same lines, ICH ADAPT2 found that the number and volume of new DWI lesions were not affected by the intensity of blood pressure control after ICH, further confirming that more aggressive blood pressure control is unlikely to result in de novo ischemia or infarction.

These landmark trials are summarized in Table 1.

### 2.4. Initial Choice of Antihypertensives

To maintain sustained blood pressure control with minimal variability, many patients admitted to the neurological intensive care unit are initiated on continuous infusion of an antihypertensive agent.

Typically, in treatment of hypertensive emergency without ICH, patients may receive continuous IV antihypertensive infusion of nicardipine, clevidipine, or nitroprusside. While nitroprusside is a potent antihypertensive, it should be avoided due to its veno-dilatory effects, increasing cerebral blood volume, and potentially worsening ICP. Compared to nicardipine, clevidipine has a faster onset, shorter duration of action, and smaller volume of infusion; however, it is more costly and can lead to more rebound hypertension when discontinued. Several recent retrospective studies comparing the efficacy of the two have shown no significant difference in time to goal BP [36,37].

One study has shown that early initiation of oral antihypertensives within 24 h of the event reduces ICU length of stay and hospital cost for patients with hypertensive ICH [6]. Furthermore, given that the results of INTERACT, INTERACT2, and ATACH2 show the safety of early aggressive SBP control, once enteric access is obtained, it is reasonable to initiate oral hypertensives.

**Figure 3:**
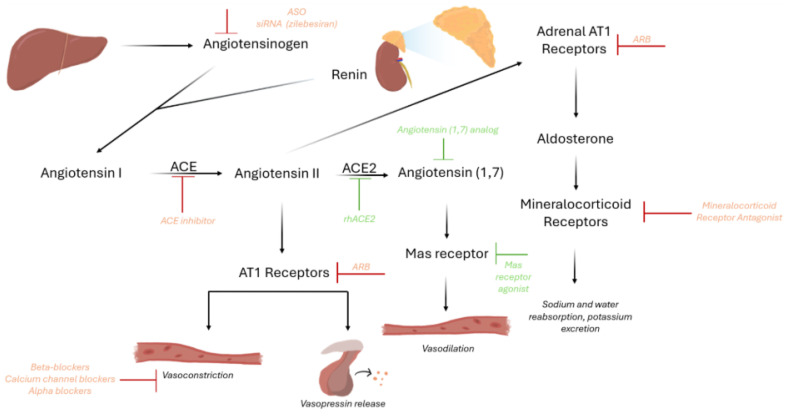
The renin–angiotensin–aldosterone system (RAAS) and the effects of current and novel antihypertensives on its modulation. Agonists or analogs are indicated in green, with inhibitors and antagonists indicated in red. ACE: angiotensin-converting enzyme; ARB: angiotensin receptor blocker; ASO: antisense oligonucleotides; rhACE2: recombinant human ACE2; siRNA: short interfering RNA.

Based on the 2025 AHA guidelines on the management of high blood pressure in adults, first-line antihypertensive therapy should be an angiotensin-converting enzyme inhibitor (ACEi) or angiotensin receptor blocker (ARB), a calcium channel blocker, and a thiazide-like diuretic (chlorthalidone or indapamide). In general, in patients with ICH, thiazide-like diuretics have been avoided in the acute period due to the potential for causing or worsening hyponatremia and worsening cerebral edema. Therefore, a mineralocorticoid receptor antagonist (MRA; chiefly, spironolactone) should be added as the next-line agent after ACE/ARB and CCB. PATHWAY-2, a randomized, double-blind crossover trial studied the impact of spironolactone vs. doxazosin, bisoprolol, or placebo in patients aged 18–79 years old with resistant hypertension in the UK and demonstrated that spironolactone was the most effective add-on agent [38]. If there are contraindications to MRA, other reasonable next-line agents include amiloride, beta-blockers, alpha-blockers, central sympatholytics, dual endothelin receptor antagonists, or direct vasodilators. The mechanisms of action of current and novel therapeutics are summarized below and in Figure 3.

## 3. ACEi/ARB

ACEi and ARBs inhibit activation of the RAAS signaling cascade and, therefore, may also prevent secondary neuronal death following ICH and inhibit atherosclerotic processes [39]. Contraindications to ACEi include angioedema (regardless of relation to prior ACE inhibitor or ARB), pregnancy, and renal artery stenosis. ACEi are teratogenic in the second and third trimester of pregnancy. Cough may occur in 5 to 20% of patients [40]. ACEi and ARBs are commonly held in patients with acute renal injury in inpatient settings. However, in patients with chronic kidney disease (CKD), the use of ACEi in patients with creatinine up to 3.0 mg/dL is considered safe. In fact, no level of creatinine is an absolute contraindication [40]. In patients with diabetes and CKD, ACEi and ARBs are recommended by the 2025 AHA guidelines as first-line agents, owing to their nephroprotective effects and the reduction in urinary albumin excretion. Based on the results of the HOPE trial, the ACEi ramipril significantly reduced rates of death, MI, and stroke in diabetic patients aged 55 or older with at least one other cardiac risk [41]. In one study comparing ACEi and ARBs, there was no significant difference in efficacy; however, ARBs generally had a lower risk of GI bleed, pancreatitis, angioedema, and cough [42]. In patients with non-lobar ICH, ACEi/ARB have been associated with improved 90-day functional outcomes, as measured by mRS and the Barthel Index, compared to other classes of antihypertensives, independent of blood pressure control [30]. When further stratified, ARBs showed statistically significant favorable outcomes, while ACEi did not [30].

## 4. Calcium Channel Blockers (CCB)

Calcium channel blockers prevent calcium influx by binding to L type voltage-gated calcium channels in vascular smooth muscle, the heart, and the pancreas [43]. The non-dihydropyridine CCBs, diltiazem and verapamil, inhibit the SA and AV nodes and reduce cardiac conduction and contractility [43]. Verapamil and diltiazem are CYP3A isoenzyme inhibitors, and caution should therefore be taken when combined with statins, benzodiazepines, buspirone, sildenafil, and cyclosporin. Due to the potential for worsening bradycardia and cardiac output, non-dihydropyridines are contraindicated in HFrEF, second- or third-degree AV block, and sick sinus syndrome. Dihydropyridine CCBs (amlodipine, felodipine, nimodipine, nicardipine, and nifedipine) have minimal cardiac binding and mostly promote peripheral vasodilation; they are most useful in post-ICH vasospasm, hypertension, and migraine. In part due to its longer half-life (30–50 h vs. 2 h) and slower onset of action (6–12 h vs. 30–60 min), amlodipine is preferred to nifedipine, as it has demonstrated a higher rate of adherence, higher absolute and relative therapeutic coverage, more consistent BP control, and lower adverse events and treatment withdrawal [44]. Especially when concern exists for adherence, amlodipine has shown greater efficacy in an overall BP-lowering effect, as it has a higher plasma concentration compared to that of nifedipine after 24 h (79% vs. 30%) and after 72 h (61% vs. 25%) [45]. Unlike nifedipine, amlodipine can be crushed, which is important for patients with ICH and dysphagia.

## 5. Thiazides

Thiazide and thiazide-like diuretics inhibit sodium reabsorption by blocking the sodium-chloride channel in the distal convoluted tubule of the nephron [46]. Thiazide diuretics include hydrochlorothiazide (HCTZ), chlorothiazide, and methyclothiazide. Thiazide-like diuretics include indapamide, metolazone, and chlorthalidone. Adverse effects include hypokalemia, hyponatremia, metabolic alkalosis, hypercalcemia, hyperglycemia, and hyperuricemia. In high doses, thiazides may cause hyperlipidemia. Given the risk of hyponatremia and possibility of worsening cerebral edema, thiazides and thiazide-like diuretics have limited utility in the acute treatment of hypertension in patients with ICH.

## 6. Mineralocorticoid Receptor Antagonists (MRAs)

Mineralocorticoid receptors, in addition to mediating the effects of aldosterone in the kidneys, have a role in extra-renal signaling, with downstream effects that may affect blood pressure regulation. These include activation of the sympathetic nervous system, endothelial dysfunction, and stimulation of vascular smooth muscle cells [47]. As primary aldosteronism is a frequent cause of secondary hypertension [47], and patients with resistant hypertension commonly develop secondary aldosteronism because of diuretic-induced sodium depletion and subsequent RAAS activation, MRAs are a good option for the next-line treatment of hypertension. Given that MRAs can result in impaired potassium excretion, this class of antihypertensives should be avoided in patients with serum potassium levels higher than 5.5 mmol/L. As discussed previously, PATHWAY 2 demonstrated that spironolactone was the most effective add-on agent compared to other classes of anti-hypertensives. The results of PATHWAY2 suggested that the benefits of spironolactone seem to be associated with a reduction in baseline plasma renin levels, supporting the hypothesis that increased sodium retention leads to resistant hypertension.

## 7. Amiloride

Amiloride is a potassium-sparing diuretic that inhibits epithelial sodium channels in the distal nephron, lung, and colon. In the distal convoluted tubule and cortical collecting duct, amiloride decreases potassium, hydrogen, calcium, and magnesium secretion, and may result in mild natriuresis. Prolonged use may reduce the excretion of uric acid. While the AHA does not recommend amiloride as an initial agent for the management of hypertension, it is approved by the FDA to be used in conjunction with thiazides for the treatment of CHF or hypertension to improve hypokalemia [48]. In an open-label, blinded endpoint randomized clinical trial in South Korea, amiloride was non-inferior to spironolactone in lowering home blood pressure [49].

## 8. Beta-Blockers

Beta-blockers function by antagonizing B1 and B2 adrenoreceptors in the heart and smooth muscle to decrease chronotropic and ionotropic effects, which in turn decreases the heart rate, blood pressure, and cardiac output [50]. There are two main beta-blocker groups: non-selective beta-blockers (e.g., propranolol), which bind to both B1 and B2 receptors, and B1-selective beta-blockers (atenolol, metoprolol, and esmolol), which only bind to B1. Carvedilol and labetalol are considered alpha and beta dual-receptor blockers, and bind to B1-, B2-, and alpha-1 receptors. The use of beta-blockers as monotherapy for hypertension is not generally recommended, as other options, such as CCB, ACE-I, and ARB, have shown greater benefits [51,52]. However, they cause a substantial reduction in blood pressure when combined with drugs of different mechanisms and are thus recommended as next-line agents. A meta-analysis from 2023 showed that adding a beta-blocker to a non-beta-blocker monotherapy significantly decreased both systolic and diastolic blood pressure; the results were consistent across different non-beta-blocker classes (ACEi/ARBs, CCBs, diuretics) [53]. In one study of 138 hypertensive ICH patients in Canada, atenolol significantly reduced mortality, rates of SIRS, and pneumonia, but did not show statistically significant improvement in outcomes at 90 days, compared to those who did not receive atenolol [54].

## 9. Alpha-Blockers

Alpha-1 adrenergic receptor blockers prevent the binding of norepinephrine to receptors on prostatic smooth muscle and vascular smooth muscle. Diminished norepinephrine binding to alpha-1 receptors results in vasodilation, which decreases peripheral vascular resistance, and ultimately lowers blood pressure [55]. Commonly prescribed alpha-blockers include doxazosin, terazosin, and prazosin.

Based on the ALLHAT hypertension trial, compared to chlorthalidone, doxazosin had a statistically significant increase in decompensated CHF, angina, and stroke; it is therefore not typically used as a first-line antihypertensive [56]. Alpha-blockers are still used as second- or third-line agents, particularly in patients with co-morbid benign prostatic hyperplasia.

In one meta-analysis of 1496 publications evaluating the dose-related blood pressure-lowering efficacy of alpha-blockers, the blood pressure-lowering effects of doxazosin and terazosin were similar [57]. The effect appears modest, on average lowering SBP by approximately 5 to 8 mm Hg [57].

## 10. Central Sympatholytics—Alpha-2 Adrenergic Receptor Agonists

Activation of the centrally located alpha-2a and -2c receptors (locus ceruleus) produce sedation, analgesia, and sympatholytic effects. Alpha-2b receptors are found most often on vascular smooth muscle and agonism of these receptors results in vasopressor effects.

The most commonly used alpha-2 receptor agonist in the management of hypertension is clonidine. Other medications in this class include guanfacine, methyldopa, guanabenz, moxonidine, rilmenidine, and dexmedetomidine [58].

Clonidine is an alpha-2 agonist, available in patch and pill formulation. Abrupt cessation precipitates rebound hypertension within 36 to 72 h of discontinuation and, therefore, is not an ideal agent in patients with poor adherence to medical therapy [58]. It can be useful in the management of ICH patients with hypertension who exhibit concomitant, paroxysmal, sympathetic hyperactivity or hyperactive delirium.

Dexmedetomidine is a selective alpha-2 adrenergic agonist used primarily for sedation. At low doses, it remains selective for central and peripheral alpha-2 receptors, and results in sedation, decreased heart rate, and systemic vascular resistance, thereby reducing blood pressure. Bolus or high-dose infusions diminish alpha-2 receptor selectivity, and result in activation of peripheral alpha-1 and alpha-2b receptors causing systemic hypertension. Caution should therefore be exercised when using high doses of dexmedetomidine as a sedative for patients with ICH [58].

## 11. Dual Endothelin Receptor Antagonists

Endothelin-1 mediates vasoconstriction through its action at endothelin-A and endothelin-B receptors. Aprocitentan is a dual endothelin-A and -B antagonist that was approved by the FDA in 2024 for the treatment of hypertension. In a multi-center, blinded, randomized, parallel-group phase 3 study, Parallel-Group, Phase 3 Study with Aprocitentan in Subjects with Resistant Hypertension (PRECISION), aprocitentan was superior to placebo in lowering blood pressure by about 4 mm Hg, at a dose of 12.5 and 25 mg daily, with sustained effects at week 40. It has a long half-life of about 44 h and may, therefore, be helpful in patients where adherence is a concern [59].

## 12. Direct Vasodilators

Direct vasodilators, such as hydralazine and minoxidil, reduce blood pressure by reducing peripheral vascular resistance. This reduced peripheral resistance is detected by arterial baroreceptors, often resulting in compensatory tachycardia.

Hydralazine is thought to promote vasodilation by inhibiting calcium release from the sarcoplasmic reticulum, which prevents myosin phosphorylation and smooth muscle contraction. Hydralazine is typically dosed three to four times daily and is, therefore, not an ideal agent if adherence is a concern.

Minoxidil activates adenosine triphosphate-modulated potassium channels in vascular smooth muscle, resulting in reduced concentration of intracellular potassium and smooth muscle relaxation. When combined with a beta-blocker and diuretic, it has been shown to be an effective agent in lowering blood pressure in those with severe or refractory hypertension. Some studies have shown that minoxidil may also be helpful in preventing the progression of renal disease. It can be dosed once daily or divided into multiple doses if the patient has low supine diastolic blood pressure [60].

## 13. Nitrates

Nitrates cause vasodilation through the donation of nitric oxide, as previously discussed. This class of drugs includes nitroglycerin, isosorbide mononitrate, and isosorbide dinitrate. These are typically used in the treatment of angina and can be used as an adjunct in the treatment of hypertension, particularly isosorbide mononitrate. It should be noted that nitrates are not FDA-approved for the management of hypertension and are not recommended as first-line therapy for hypertension [7]. However, isosorbide dinitrate, in combination with hydralazine, is recommended to reduce afterload in HFrEF, particularly in Black patients [7]. Due to the potential for causing severe hypotension, nitrates should not be used in conjunction with sildenafil, tadalafil, vardenafil, and avanafil.

## 14. Future Directions

Whether related to the clinical management of resistant hypertension in ICH or the elucidation of its molecular mechanisms, several promising therapeutic targets and technological advances are on the horizon.

## 15. Novel Therapeutics

### 15.1. Antisense Oligonucleotides and Small Interfering RNAs

Novel therapeutics aim to silence the hepatic production of angiotensinogen [61] using antisense oligonucleotides (ASOs) and small interfering RNAs (siRNAs). At present, there are two RNA therapeutics in development: IONIS-AGT-LRx and ALN-AGT, also known as zilebesiran. Both are administered with subcutaneous injections and are conjugated to N-acetylgalactosamine (GalNAc) for targeted delivery to hepatocytes. A randomized, placebo-controlled first-in-human/phase I trial with zilebesiran showed a profound and sustained reduction in SBP by up to 20 mmHg over a period of 24 weeks and after a single dose of the drug [62]. Although not powered to detect an effect on blood pressure, the phase 2 trials for IONIS-AGT-LRx did not show a significant blood pressure reduction, likely due to insufficient angiotensinogen gene silencing [63]. As such, at this stage, zilebesiran appears to have a more robust blood pressure-lowering effect than IONIS-AGT-LRx. Both were shown to be well tolerated without incidences of hyperkalemia or AKI.

### 15.2. Recombinant Human ACE2, Angiotensin (1,7), and the Mas Receptor

ACE2, which hydrolyzes angiotensin II to angiotensin (1,7), is hypothesized to have protective effects in lung injury and cardiovascular disease. Angiotensin II acts on AT1 receptors, ultimately resulting in vasoconstriction and aldosterone release. Therefore, enhanced breakdown of angiotensin II may lead to lower blood pressure. In one in vivo study of male mice, Wysocki et al. [64] showed that recombinant human ACE2 effectively degraded Ang II and improved blood pressure. The activity of angiotensin (1,7) at Mas receptors has been shown to increase nitric oxide production in cardiomyocytes, thereby mediating vasodilation. While the promotion of ACE2 activity, angiotensin (1,7), and Mas receptor activity has been shown to be effective in mouse studies, unfortunately, activation of this pathway has thus far been unfruitful beyond phase I trials [61].

### 15.3. Extracellular Vehicles (EVs) and miRNA

EVs are spherical lipid bilayer vesicles secreted by most cells of the body that carry genetic material and proteins, enabling cells and tissues to communicate over short and remote distances [65,66]. They act as important biomarkers of disease, utilized both for their diagnostic and therapeutic properties. Depending on the nature of the stimulus that promotes their release, the content of their cargo and the target tissue, EVs can be pathogenic or physiologic. For instance, in hypertensive states, platelets and endothelial cells increase their release of specific microRNAs (miRNA or mIR), which are small, non-coding RNA molecules that regulate gene expression in recipient cells. MiR-143 and 145 production in VSMC is increased and their upregulation exacerbates Ang II-mediated effects on blood pressure. Mice knockouts of mIR-143/145 experience a reduced effect of Ang II-induced hypertension and a boosted reduction in blood pressure in response to ACE inhibitors [67,68]. Silencing mIR-143 and 145 offer, therefore, a potential therapeutic avenue for resistant hypertension. Another example of miRNA with potential therapeutic benefits is mIR-29a/b-3p which, when levels are increased, reduce lysophospholipase-1, an enzyme that inhibits the activity of endothelial nitric oxide synthase (eNOS) activity and NO production [69]. Therefore, enrichment with mIR-29a/b-3p increases NO production, improving vascular endothelial function and lowering blood pressure.

### 15.4. Gut Microbiome Modulation

Although it has shown promise in animal models of hypertension, modulating the gut microbiome has yet to show promise in clinical studies. The first randomized fecal matter transplantation trial in humans showed a significant but non-sustained blood pressure-lowering at week 1 of 4.34 mmHg (95% CI, −8.1 to −0.58; *p* = 0.024) [70]. Other meta-analyses of prebiotics and probiotics in the management of hypertension have only shown very modest blood pressure-lowering effects [71,72]. Microbial metabolites may be more effective in treating hypertension in humans. Short-chain fatty acids (SCFAs), such as acetate, butyrate and propionate, are the end-product of fermentation of dietary fibers and starches by commensal bacteria [73]. They enhance the gut homeostasis and gut epithelium barrier through: (1) activating inflammasomes; (2) stimulating B cells to produce secretory IgA antibodies; (3) stimulating the production of regulatory T cells (T-reg), which produce the anti-inflammatory cytokines TGF-beta, IL-10; and (4) increasing mucus production by goblet cells. Their actions on the CNS aim to decrease neuroinflammation by: (1) increasing vagal signaling to the brain through enhanced production of neurotransmitters and neuropeptides (serotonin, gamma-aminobutyric acid, PYY, GLP1 and 2; (2) reinforcing tight junctions, thereby promoting blood–brain barrier (BBB) integrity; and (3) modulating microglial development and function, shifting their polarity from a pro-inflammatory M1 phenotype to a restorative/reparative M2 phenotype [74]. In a phase 2, double-blinded, crossover study of 20 patients with untreated hypertension, SBP at 24 h was lowered by a mean of 6.1 mmHg when patients were treated with HAMSAB, a type of starch that releases acetate and butyrate in the colon, compared to when they were treated with placebo [75].

## 16. Unanswered Questions

Several areas in the management of resistant hypertension in ICH remain unclear. One of these is the parameter best suited to be a goal of therapy. While SBP has been traditionally the strongest and most commonly referenced blood pressure parameter in the management of hypertension, it may not be the only important value to enter into consideration. In fact, blood pressure variability (BPV) is increasingly recognized as an independent and critical variable with a significant impact on end-organ damage and cardiovascular risk [76]. However, the guidelines remain vague as to the method of choice for monitoring and limiting BPV post ICH.

Another question of clinical relevance is the optimal blood pressure target in patients with resistant and refractory hypertension after an ICH. This question is also linked to the optimal timeline for achieving these unknown targets. Defining these goals becomes critical in the presence of severe comorbidities such as cerebral, coronary, or renal vascular disease.

## 17. Workup of Secondary Hypertension

Resistant hypertension should be differentiated from refractory hypertension (defined as elevated blood pressure despite the use of five or more antihypertensives), and apparent resistant hypertension due to nonadherence, white coat syndrome, or improper blood pressure measurement, should be excluded. Thereafter, workup for secondary causes of hypertension should be pursued. Once the diagnosis of resistant hypertension has been established, workup of secondary hypertension should be pursued. Patients should be screened for sleep disorders (sleep apnea, restless leg syndrome, etc.), primary aldosteronism, renal parenchymal disease, renal artery stenosis, pheochromocytoma/paraganglioma, and endocrine disorders [11].

Fragmented, poor-quality sleep contributes to elevated blood pressure through heightened sympathetic activity and dysfunction of the RAAS. In patients with obstructive sleep apnea, increased adherence to CPAP therapy modestly and proportionately reduces blood pressure (average 2 to 5 mmHg). Adjunctive antihypertensives are typically required. Polysomnography may not be required in all patients, but patients should be screened for signs and symptoms of OSA.

Primary aldosteronism is a common cause of resistant hypertension, comprising approximately 20% of patients with resistant hypertension. Compared to patients with primary hypertension, patients with primary aldosteronism have higher risks of cardiovascular disease (4.2-fold increased risk of stroke, 6.5-fold increased risk of myocardial infarction, and 4.2-fold increased risk of atrial fibrillation), owing to toxic effects of aldosterone on the heart and vessels. Screening may be performed by checking the plasma aldosterone to renin ratio (ARR), obtained in the morning with the patient seated for at least 30 min prior to sampling. Confirmatory testing should be performed if screening is positive, i.e., the ARR is greater than 20–30.

Renal ultrasound and doppler may be used to evaluate renal parenchymal disease and renal artery stenosis. Renal parenchymal disease, as with chronic kidney disease, contributes to resistant hypertension through impairment of sodium excretion and fluid retention. CKD may also occur as a consequence of untreated hypertension. Renal artery stenosis most commonly occurs because of atherosclerotic disease, but may occur with Takayasu arteritis, fibromuscular dysplasia, or radiation. Chronic renal ischemia from renal artery stenosis results in activation of the RAAS, thereby worsening hypertension.

Pheochromocytoma and paraganglioma are rare but important causes of resistant hypertension, manifesting as paroxysmal episodes of hypertension. Diagnosis of these tumors is often delayed by 3 years, and up to one third of cases are inherited. Screening may be done by measuring circulating catecholamine metabolites: plasma free or urinary fractionated metanephrines. Patients with essential hypertension may also have elevations of metanephrines. Therefore, these studies should be repeated if elevated on initial screening. This is often followed by imaging to detect the tumors, using a combination of CT, MRI, and nuclear scans such as MIBG (metaiodobenzylguanidine) and PET scans.

Endocrine disorders such as thyroid disease (hyper and hypothyroidism), hyperparathyroidism, congenital adrenal hyperplasia, and acromegaly may also contribute to resistant hypertension. Screening may be performed with TSH/T4/T3, along with measurement of serum calcium, potassium, renin, and aldosterone. If there is high clinical suspicion, patients with acromegaly commonly have elevated serum insulin-like growth factor-1 (IGF-1) levels and may also have elevated serum growth hormone levels that fail to suppress with oral glucose load.

When diagnosed or suspected, consultation with Nephrology, Cardiology, Endocrinology and/or Rheumatology may be required to complete the workup and initiate appropriate therapies.

## 18. Considerations for the Long-Term Management of Resistant Hypertension

Patients with resistant hypertension have a higher risk of poor outcomes compared to those without. They are 47% more likely to have myocardial infarct, heart failure, stroke, CKD, or death at a median 3.8 years of follow up [11]. Persistently elevated 3-month blood pressure measurements in survivors of ICH are associated with higher recurrent stroke risk and mortality [8]. Therefore, care should be taken to optimize a patient’s antihypertensive regimen to ensure adherence to medical therapy. This can be achieved by selecting medications with less frequent dosing (once or twice daily) and by choosing combination pills, when available [7].

Artificial intelligence offers an exciting avenue with which to encourage compliance with at-home monitoring of blood pressure [77]. In one study of 2000 patients, participants were contacted using a voice-enabled AI agent and guided through a live blood pressure measurement or to report a recent blood pressure measurement. These data were subsequently validated and submitted into the EHR for review by a clinician; care management referrals were then triggered in cases of uncontrolled blood pressure. Patient-reported satisfaction exceeded 9 out of 10, and improved compliance with MA Stars Controlling Blood Pressure HEDIS benchmarks by 17%. The investigators demonstrated a cost-effective way to enhance patient engagement and adherence to therapy while reducing clinician burden.

Lifestyle changes, including regular exercise and weight loss, should be encouraged. Treadmill walking three times weekly for 8 to 12 weeks has been shown to significantly lower daytime ambulatory BP in patients with resistant hypertension [78]. A body mass index greater than 30 kg/m^2^ is an independent risk factor for resistant hypertension [11]. Patients should be counseled to follow a heart-healthy diet (DASH diet, reduce sodium intake, and eliminate alcohol intake).

Once discharged from the hospital, patients should follow up regularly with their primary care physician, neurocritical care team, and vascular neurology. Many patients in this population have not previously established care with a primary care physician; this is paramount. As much of their follow-up care relates to the condition for which they were admitted to the critical care unit rather than other primary medical illnesses, continued care with the critical care team in the clinic setting is an important adjunct [79]. There is a paucity of studies in the literature on the optimal frequency of follow up in this population; further research on the topic should be pursued.

## 19. Conclusions

**Figure 4:**
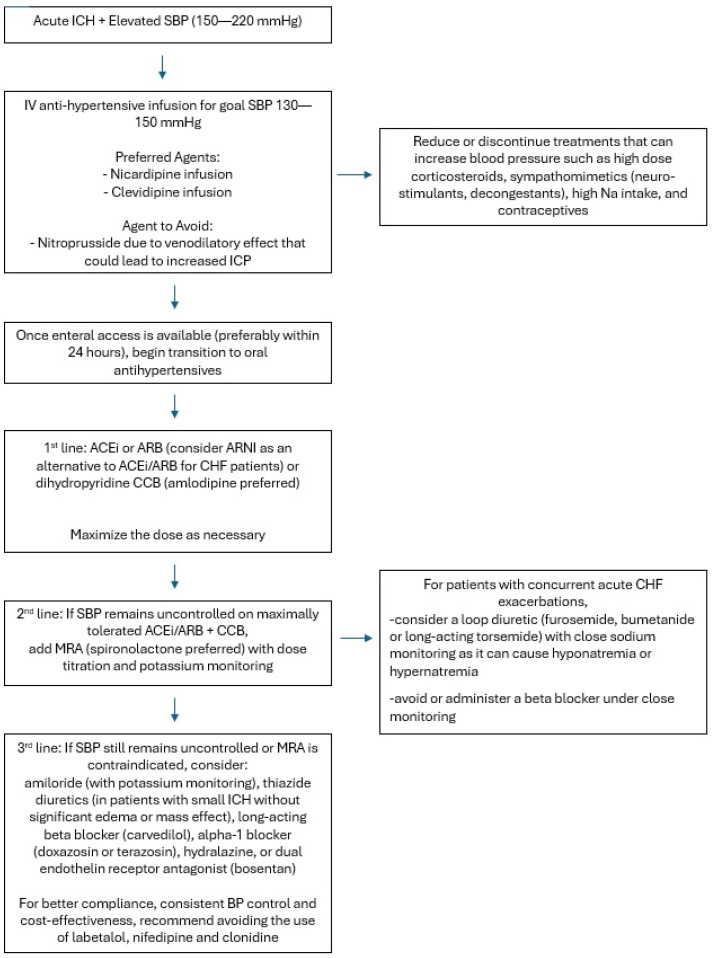
Proposed algorithm for the optimal pharmacological management of resistant hypertension in ICH.

The adequate control of blood pressure remains a cornerstone of treatment in ICH. An early reduction in SBP, to target 130–150 mm Hg with smooth, sustained BP control, may improve functional outcomes in patients with mild to moderate ICH. After initiating oral antihypertensives with a calcium channel blocker and an ACEi/ARB to maximally tolerated doses, the next-line antihypertensive should be tailored to the patient’s co-morbid conditions. Spironolactone offers an underutilized opportunity for effective blood pressure control and should be considered early in the management of resistant hypertension in patients with ICH. Based on the 2025 AHA guidelines and the findings of PATHWAY-2, we propose a simple algorithm for the optimal pharmacological management of resistant hypertension in ICH in Figure 4. Once resistant hypertension is identified, workup for secondary hypertension should be pursued in addition to referrals to appropriate specialists.

Current research into mitigating secondary brain injury after an ICH focuses on three primary areas: cellular death pathways, neuroinflammatory responses, and the influence of the gut–brain axis in high-risk hypertensive patients. Advancements in hypertension management are shifting toward genetic silencing (ASOs and siRNAs) and AI-driven monitoring to enhance therapeutic precision and patient adherence.

Significant gaps persist in treating resistant hypertension, specifically regarding the standardized management of blood pressure variability (BPV) and the lack of clear guidelines for patients with extreme hypertension (>220 mmHg) or complex comorbidities. Addressing these nebulous areas is vital for establishing a definitive standard of care for high-risk post-ICH populations.

## Figures and Tables

**Table 1:** A summary of the major findings from landmark trials in ICH: ATACH II, INTERACT 1, 2, 3, and 4 [31,32,33,35].

Trial	Timing	Intervention	Major Findings	Safety Outcomes
INTERACT 1 (2008)	ICH diagnosed within 6 h of onset	Intensive: SBP < 140 mmHg Standard: SBP < 180 mmHg	Although not statistically significant, relative risk of hematoma growth was lower in the intensive group than in the standard group (*p* = 0.05).	No significant difference in the risks of adverse events between the two groups
INTERACT 2 (2013)	ICH diagnosed within 6 h of onset	Intensive: SBP < 140 mmHg Standard: SBP < 180 mmHg	The intensive group had lower modified Rankin scores compared to the standard group (*p* = 0.04).	No significant difference in mortality and nonfatal serious adverse events between the two groups
ATACH II (2016)	ICH diagnosed within 4.5 h of onset	Intensive: SBP < 110–139 mmHg Standard: SBP 140–179 mmHg	No significant difference in primary outcome of death or disability between the two groups	No significant difference in serious adverse events between the two groups The rate of renal adverse events within 7 days was significantly higher in the intensive group compared to the standard group (*p* = 0.002).
INTERACT 3 (2023)	ICH diagnosed within 6 h of onset	Care bundle: target SBP < 140 mmHg, strict glucose control, anti-pyrexia and rapid reversal of warfarin-related anticoagulation Usual care: varied depending on hospital practice	The care bundle group had lower likelihood of poor functional outcome compared to the usual care group (*p* = 0.015).	The care bundle group had fewer serious adverse events compared to the usual care group (*p* = 0.0098).
INTERACT 4 (2024)	Presumed acute stroke with elevated SBP (150 mmHg or higher) with the ability to start treatment within 2 h of symptom onset or last known well	Intervention group: SBP 130–140 mmHg within 30 min until arrival at the hospital Usual care group: SBP < 220 mmHg or DBP < 110 mmHg	No significant difference in functional outcome between the two groups	No significant difference in the incidence of serious adverse events between the two groups

## Data Availability

No new data were created or analyzed in this study. Data sharing is not applicable to this article.

## Abbreviations

ACE	angiotensin-converting enzyme
ACEi	angiotensin-converting enzyme inhibitor
AHA	American Heart Association
AI	artificial intelligence
ATACH II	Antihypertensive Treatment of Acute Cerebral Hemorrhage II
Ang I	angiotensin I
Ang II	angiotensin II
ARB	angiotensin receptor blocker
ARR	aldosterone to renin ratio
ASOs	antisense oligonucleotides
AT1R	angiotensin II type 1 receptor
BBB	blood–brain barrier
BP	blood pressure
BPV	blood pressure variability
CCB	calcium channel blocker
CKD	chronic kidney disease
CPAP	continuous positive airway pressure
DEGs	differentially expressed genes
ECM	extracellular matrix
GalNAc	N-acetylgalactosamine
GSH	glutathione
GPX4	glutathione peroxidase 4
HFrEF	heart failure with reduced ejection fraction
ICH	intracerebral hemorrhage
ICU	intensive care unit
IGF-1	insulin-like growth factor 1
INTERACT trial	Intensive Blood Pressure Reduction in Acute Cerebral Haemorrhage Trial
KEGG	Kyoto Encyclopedia of Genes and Genomes
MI	myocardial infarction
MIBG	metaiodobenzylguanidine
MRAs	mineralocorticoid receptor antagonists
mRS	Modified Rankin Scale
NO	nitric oxide
NO-NOsGC-cGMP	nitric oxide–nitric oxide sensitive guanylate cyclase—cGMP pathway
OSA	obstructive sleep apnea
PET	positron emission tomography
PRES	posterior reversible encephalopathy syndrome
PVN	paraventricular nucleus (of the hypothalamus)
RAAS	renin–angiotensin–aldosterone system
RCT	randomized control trial
ROS	reactive oxygen species
SBP	systolic blood pressure
SCFAs	short-chain fatty acids
siRNAs	small interfering RNAs
TLR4	toll-like receptor 4
TSH	thyroid-stimulating hormone
VSMCs	vascular smooth muscle cells
